# Statement of Retraction

**DOI:** 10.1080/23802359.2017.1343535

**Published:** 2017-06-29

The following article has been retracted from publication in the Taylor & Francis journal

Mitochondrial DNA Part B: Resources:

Juanjuan Liu, Chun Tan, Kan Xiao, Binzhong Wang, Xueqing Liu and Hejun Du. 2017. The complete mitochondrial genome of Dabry’s sturgeon (Acipenser dabryanus), Mitochondrial DNA Part B: Resources, 2:1, 54–55 http://dx.doi.org/10.1080/23802359.2017.1280698

This article has been retracted at the request of the authors, as subsequent to publication it has been found that the data reported in the article contained serious errors.

Taylor & Francis note we received, peer-reviewed, accepted, and published the article in good faith based on warranties made by the authors.

The retracted article will remain online to maintain the scholarly record, but it will be digitally watermarked on each page as “Retracted”.

